# A Picky Predator and Its Prey: How Snow Conditions and Ptarmigan Abundance Impact Gyrfalcon Feeding Behaviour and Breeding Success

**DOI:** 10.1002/ece3.71228

**Published:** 2025-04-09

**Authors:** Annabel Josien Slettenhaar, Jan Eivind Østnes, Børje Cato Moen, Rolf Terje Kroglund, Torgeir Nygård, Erlend Birkeland Nilsen

**Affiliations:** ^1^ Faculty of Bioscience and Aquaculture Nord University Steinkjer Norway; ^2^ Naturporten Nordli Norway; ^3^ Norwegian Institute for Nature Research Trondheim Norway

**Keywords:** climate change, *Falco rusticolus*, functional response, *Lagopus* spp., numerical response, predator–prey dynamics

## Abstract

Species interactions can be altered by climate change but can also mediate its effects. The gyrfalcon ( *Falco rusticolus* ) and the ptarmigan (*Lagopus* spp.) form a predator–prey couple that reflects the dynamics of boreal, tundra, and alpine ecosystems. To determine how climate change may impact the alpine food web, we investigated how ptarmigan abundance and local weather impact gyrfalcon diet and feeding behaviour, nest occupancy, and reproductive success. Using wildlife cameras, we monitored gyrfalcon nests throughout the nestling period to collect data on diet and feeding behaviour. We quantified the gyrfalcon's functional response by describing how ptarmigan kill rates relate to ptarmigan density. Additionally, we quantified the gyrfalcon's numerical demographic and aggregative response by describing how gyrfalcon reproductive success and nest occupancy, respectively, were related to ptarmigan density, using data from large‐scale monitoring projects. Ptarmigan were the dominant prey species, representing 98% of the diet. The proportion of ptarmigan in the gyrfalcon diet and gyrfalcon breeding success increased in springs with more snow, but breeding success decreased with more snow during the nestling period. Gyrfalcon reproductive success was positively related to ptarmigan density, but gyrfalcon nest occupancy and the ptarmigan kill rate were not related to ptarmigan density. These results indicate that the effect of climate change is not straightforward, and investigating how (a)biotic factors impact both prey and predator is relevant in predicting how a predator will respond to climate change. Following current climate predictions, spring will occur earlier, which will change the food‐web structure through prey availability and diversity and through interactions with other species. This requires adaptations from gyrfalcons and other predators. We emphasise that the impact of climate change on predators and other species can be more accurately evaluated on a multi‐species level rather than individually.

## Graphical abstract

This study was conducted in the alpine areas of central Norway, and focused on the gyrfalcon (*Falco rusticolus*) and two ptarmigan species (*Lagopus lagopus*, *Lagopus muta*). We provide a detailed description of gyrfalcon diet throughout the nestling period, describe how this, reproductive success and territory occupancy relate to local ptarmigan densities, and what the effect is of spring timing and local weather variation. We show that advancing onset of springs could pose a threat for the gyrfalcon in the future, but that accurate predictions of climate change effects require a multi‐species approach.

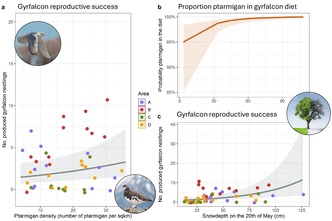

## 1 Introduction

Species interactions are at the foundation of ecosystem functioning, and they can play a role in mediating the impact of climate change on biodiversity (Åkesson et al. 2021; Alexander et al. 2015). Increasing temperature and changes in weather and precipitation patterns due to climate change (IPCC 2023) threaten biodiversity and ecosystem functions (Scheffers et al. 2016; Trew and Maclean 2021; Zhang et al. 2013). Several studies have reported that environmental change can significantly alter food‐web dynamics and predator–prey interactions (Bestion et al. 2019; Petchey et al. 1999). Predators may be forced to adjust their diet and switch to alternative prey when a prey species declines throughout their distribution (Winfield et al. 2012). A local decline of top predators can cause a so‐called trophic cascade, altering food‐web dynamics and increasing population growth of species at lower trophic levels. Therefore, studies have argued that species interactions should be included in the framework for climate change predictions, rather than solely focusing on the direct effects of climate on a single species (Araújo and Rozenfeld 2014; Cahill et al. 2013; Gilman et al. 2010). Predator–prey interactions occur across multiple trophic levels, which makes them highly relevant to study in the context of environmental change (Allesina and Pascual 2008; Laws 2017).

Higher trophic levels are affected by climate change disproportionately, since they are also indirectly affected by changes in lower trophic levels (Voigt et al. 2003). Specialist predators are particularly vulnerable, because they rely mainly on a single prey species. Their preferred prey species can decline in numbers due to a changing environment, but the effort it takes the predator to catch that prey can also change, e.g., due to changed habitat structure impacting camouflage or shelter from vegetation (Morin et al. 2021; Zimova et al. 2020). Unlike generalist predators, specialists cannot easily switch to other prey species when the availability of their preferred prey species declines. Therefore, assessing the resilience of a specialist predator species to a changing climate involves understanding how their hunting rates and productivity are impacted by fluctuations in the prey population (Peers et al. 2014; Terraube et al. 2015). Evaluating their resilience will also contribute to our ability to anticipate future changes in the food web, given the crucial role predators play in this process.

An example of a specialist predator–prey couple is the gyrfalcon ( *Falco rusticolus* ) and the ptarmigan (willow ptarmigan *Lagopus lagopus* or rock ptarmigan *L. muta*, hereafter collectively referred to as ‘ptarmigan’), which both have a circumpolar breeding distribution. The entire breeding range of the gyrfalcon overlaps with at least one of the ptarmigan species (Nielsen and Cade 2017). Besides being specialists, gyrfalcons become more vulnerable by living at high latitudes and elevations, where climate warming is more pronounced (Brunetti et al. 2009; IPCC 2023). Their species range is a suitable environment to study predator–prey interactions in this context, because food webs tend to be relatively simple here due to lower species diversity (Gibert 2019; Paine 1966). In several studies, the importance of ptarmigan abundance for gyrfalcon populations has been demonstrated (Barichello and Mossop 2011; Hagen 1952; Nielsen 1999). Almost all studies on gyrfalcon diet and feeding behaviour show that ptarmigan are the dominant prey species and represent 50%–100% of the diet, suggesting that the gyrfalcon behaves as a specialist predator to a large extent (Booms and Fuller 2003a; Koskimies and Sulkava 2011; Robinson et al. 2019), but see Muir and Bird (1984). The same studies also show that alternative prey, such as migratory birds and small rodents, generally become more important in the gyrfalcon diet during the summer when the diversity of available prey increases. Ptarmigan are often the only available prey in their size class throughout the winter, and remain the dominant prey for gyrfalcons throughout the year.

In many strongly linked predator–prey couples, predator behaviour and population dynamics respond to changes in prey abundance and availability. A common method to quantify this relationship is to analyse the functional and numerical response (Solomon 1949). The functional response describes how prey density affects a predators' kill rates or consumption rates of a specific prey species (Abrams 2022), and can resemble one of four curve types (Holling 1966). The shape of the curve is primarily influenced by kill rate and prey density, with kill rate itself depending on the time spent searching for, handling, and processing prey. For a specialist predator, a type II response is typical (Abrams 1990; O'Donoghue et al. 1998), which describes a kill rate that increases with prey density but asymptotically approaches the predator's prey processing capacity. The numerical response can be divided into two parts, i.e., the numerical demographic response and the numerical aggregative response. The numerical demographic response describes the relationship between prey density and predator reproductive success, whereas the aggregative response describes the relationship between prey density and territory occupancy of the predator (Bayliss and Choquenot 2002; Oksanen et al. 2001). For predators that depend on a specific prey species, one would expect a positive relationship in both cases. For species that do not reach their reproductive age in their first year of life, such as the gyrfalcon, the numerical aggregative response may be delayed (Nielsen 1999).

Studies show contrasting patterns when describing the effect of a phenological shift towards warmer springs on ptarmigan and gyrfalcon reproductive success. Some show that advanced snow melt is beneficial because it leads to earlier availability of nesting sites for both species (Eriksen et al. 2023; Nielsen 2011; Wann et al. 2016; Wilson and Martin 2010). More snow‐free vegetation is also associated with higher food availability for ptarmigan, and alternative prey for gyrfalcons such as migratory bird species can arrive at their breeding grounds earlier. However, not all studies agree (Melin et al. 2020) and increased precipitation in spring could also have a negative effect on reproduction in both species (Clarke and Johnson 1992; Robinson et al. 2017). Such changes in weather can have a direct effect on gyrfalcon reproductive success by affecting nestling survival, but it may also have an indirect effect through changed hunting success and thus feeding behaviour (Kämpfer et al. 2022; Sergio 2003). Feeding behaviour and feeding rates play a crucial role in determining fledging success and therefore reproductive success (Grames et al. 2023; Olsen et al. 1998).

The aim of this study is to analyse the diet and feeding behaviour of gyrfalcons in central Norway during the breeding season and to assess their functional and numerical response to fluctuating ptarmigan abundances. Additionally, we aim to investigate how local weather variables influence diet, feeding behaviour, reproductive success, and territory occupancy. This will help us understand the potential impacts of climate change on the ecology of this predator–prey couple. Based on previous studies on this matter, we formulated the following main predictions:

1. Ptarmigans are the dominant prey species in the gyrfalcon diet throughout the breeding season, and the proportion of alternative prey increases towards the end of this period.
2. Gyrfalcons show a type II functional response, a positive numerical demographic response, and a positive numerical aggregative response with a time lag of 1–3 years.
3. In years with early snow melt, the overall amount of prey delivered is higher, which results in higher territory occupancy and breeding success.

## 2 Materials and Methods

The aims and detailed hypotheses for this study were preregistered and are available online at the Open Science Framework (Slettenhaar et al. 2023).

### 2.1 Study Area

**FIGURE 1:**
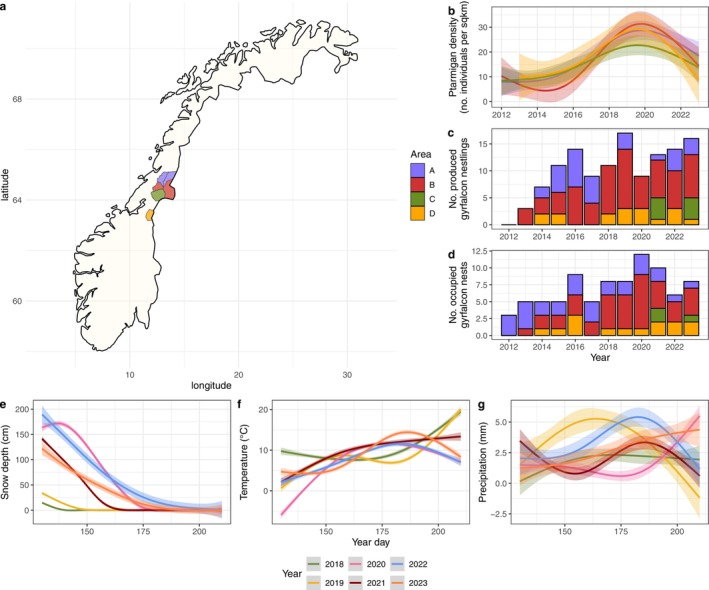
(a) The study area and the municipalities included in the study. Northernmost area A consists of municipalities Namsskogan and Røyrvik, B of Grong and Lierne, C is Snåsa, and the southernmost area D is Meråker. (b) Estimated ptarmigan densities per area as number of individuals per square kilometre (solid lines), with 95% confidence intervals (shaded ribbons). (c) The total number of gyrfalcon nestlings produced per area, i.e., combined productivity. (d) The number of occupied gyrfalcon nests per area. (e) Estimated relationships between day of the year during the nesting period and predicted snow depth, (f) temperature and (g) precipitation. Averages shown in solid lines with 95% confidence intervals (shaded ribbons).

We conducted the study in the northeastern part of central Norway. Gyrfalcon territories are found in alpine and subalpine mountain regions with a mean elevation of 667 m above sea level. The habitat is dominated by heathlands and open woodlands covered by scattered patches of mountain birch ( *Betula pubescens* ), willow (*Salix* spp.), Norway spruce ( *Picea abies* ), and Scots pine ( *Pinus sylvestris* ) (A. Moen 1999). The patchy vegetation provides food and shelter for herbivores like ptarmigan, hares ( *Lepus timidus* ), reindeer ( *Rangifer tarandus* ) and small rodents. In this area, gyrfalcons generally nest on rocky cliffs, typically in nests built by common ravens ( *Corvus corax* ). The local climate can be defined as northern or middle boreal, with temperatures around −10°C in January and snow cover persisting in the period between October and late May. Summers are relatively short, with mild temperatures fluctuating around 12°C in July (Kålås et al. 2010; Nilsen et al. 2020). See Figure 1 for the local weather conditions throughout the gyrfalcon breeding seasons during the study period.

### 2.2 Data Collection Procedures

Our analyses were based on four main data sets: (i) diet and feeding behaviour data from wildlife cameras on gyrfalcon nests, (ii) monitoring data of gyrfalcon nest occupancy and reproductive success, (iii) ptarmigan abundance data, and (iv) gridded weather data, which are each described in more detail below.

#### 2.2.1 Gyrfalcon Diet and Feeding Behaviour

We collected data on diet and feeding behaviour in Lierne municipality during the breeding seasons of 2018 through 2023. Here, we annually monitored 22 nest sites for breeding activity. On each active gyrfalcon nest, we installed two Minox DTC 550 wildlife cameras (Minox GmbH, Isny im Allgäu, Germany) when the nestlings were between 10 and 20 days old, in late May to early June, and uninstalled them in late June to early July after the nestlings fledged. The cameras recorded activity of the adults and the nestlings from two angles, enhancing our ability to observe the prey items. We programmed the cameras to take pictures with a 15 and 30 s interval after the motion sensor was triggered to ensure that each camera captured different moments of the prey deliveries. In 2018, which was our first year of camera monitoring, only one camera was installed per nest, set to a 30 s interval. Throughout the study period, we monitored a total of 12 breeding attempts at eight different nests. From the pictures, we extracted seven variables about feeding behaviour and diet of the gyrfalcons during the nesting period, every time a prey item was delivered to the nest. We determined (i) *brood size* as the number of nestlings visible in the nest. We determined (ii) *nestling age* using nestling plumage characteristics following Moen (2022). We determined (iii) *prey species* based on size, plumage, and other external features. We recorded (iv) *timing of prey delivery* and (v) *the time parents spent feeding nestlings* by recording the time passed between the first picture that feeding is visible until the last picture the parent is present moving on the nest. In gyrfalcons, females are bigger than males (Booms et al. 2020), so we determined (vi) *sex of the parent delivering the prey or feeding the nestlings* by evaluating their size when both parents were on the nest at the same time, and otherwise by recognising colour and feather patterns on the back and tail. We evaluated whether the prey item was (vii) *a fresh delivery or a cached item* from a previous delivery when we saw a partially eaten prey item return to the nest, which is a common behaviour in gyrfalcons (Booms and Fuller 2003b). We observed this behaviour more frequently early in the monitoring period when nestlings did not finish the entire prey in one feeding event.

Identifying prey items was sometimes challenging when the prey was plucked and processed before delivery to the nest. Therefore, our dataset contained a significant number of unknown prey items, 175 out of 1062 prey deliveries (16.5%). We considered three approaches to address this, which each resulted in a similar outcome. We considered (1) removing unknown prey from the dataset, (2) imputing prey type for unknown prey items using an intercept‐only model, and (3) imputing prey type using a regression model with covariates. We have chosen the latter, since this method also takes environmental context into account, and will likely lead to more reliable estimates. To do this, we estimated the probability that a prey item is a ptarmigan for each missing value, using a logistic regression model. The model included nestling age, precipitation, snow depth, and temperature as covariates (see below for a detailed description of the weather variables). Availability of prey changes throughout the nestling period, which is reflected by the covariates nestling age and snow depth, and the visibility of prey types changes with weather conditions, as reflected by precipitation and temperature. Based on these probabilities, we drew values from a binomial distribution, 1 for ptarmigan and 0 for alternative prey, to replace missing observations. We evaluated the performance of the logistic regression model by calculating Area Under the Curve (AUC). Throughout the analyses, we classified willow and rock ptarmigan collectively as ptarmigan, as they were virtually impossible to distinguish in the pictures.

#### 2.2.2 Gyrfalcon Population Monitoring

We used long‐term data on gyrfalcon reproductive success and nest occupancy from 2012 through 2023 in four areas. The areas represent a north–south gradient within the northern part of the county Trøndelag, with A being the northernmost area, and D the southernmost (Figure 1). We surveyed all nest locations known in the study area every year, and searched suitable cliffs for potential new nest sites. When new nest sites were discovered, they were added to the surveys, resulting in 64 nest locations at the end of the monitoring period. We performed the monitoring late in the nesting period in June, mostly using helicopters, which is an effective method to survey large areas for raptor activity (Olson et al. 2015). The helicopter, typically a Robinson R44 Raven, flew at a safe distance between 30 to 70 m from nests and could hover, so we could count nestlings with binoculars while disturbance is limited (Grubb et al. 2010). Helicopters always carried the same pilot and the same observer with an assistant. Digital photos of occupied nests were taken to post‐check for errors when counting nestlings from the air. To limit the costs for, and environmental impact of flying the helicopter, we surveyed more easily accessible nest locations on foot instead. We believe that nest occupancy observations from the ground are equally reliable as those from helicopters, but due to lower vantage points, nestling counts may be underestimated. We determined nest occupancy by observing the presence of territorial adults in the area, nestlings in nests or near the nesting site, or by identifying other clear signs of a breeding attempt. These signs include fresh prey remains and guano stains that are typical for gyrfalcons, i.e., purely white in colour and less massive than raven stains. Gyrfalcon reproductive success was measured as the number of nestlings present in the nest during the survey, assuming that none of them have fledged yet, and all will fledge several days later. Territory occupancy was represented by a binary variable being either occupied, or unoccupied.

#### 2.2.3 Ptarmigan Abundance

Ptarmigan populations in various regions of Norway were surveyed annually in August using line transects, as part of a monitoring program led by the Norwegian Institute for Nature Research (NINA). Data from the surveys are stored and managed in Hønsefuglportalen (https://honsefugl.nina.no/Innsyn/nb), which coordinates grouse line transect surveys across Norway. For the surveys, at least two trained observers accompanied by a dog walk along predefined transect lines, which are at least 3 km long, and are spaced at least 500 m apart. The observers count ptarmigan that are flushed along this line, while distinguishing between male and female adults and juvenile birds, and noting the perpendicular distance from the transect line at which the birds were observed. More detailed information about these surveys can be found in Bowler et al. (2020) and Kvasnes et al. (2018). Data can be accessed through the data portal of Living Norway Ecological Data Network (Nilsen et al. 2022) or via GBIF, and ingested to R using the *LivingNorwayR* package (Chipperfield et al. 2022). Using the line transect data and the distance sampling method, we estimated abundance and density of ptarmigan in the four areas shown in Figure 1. We set up a distance sampling model using the R package *Distance* (Miller et al. 2019) to estimate annual ptarmigan densities for the autumns of 2009 through 2023 for each area. In the detection model we used the half‐normal key function, a truncation distance of 200 m and cluster size, i.e., the total number of ptarmigan counted per observation, as a detection covariate. We included both rock ptarmigan and willow ptarmigan in the ptarmigan density estimates. For all but three municipalities ptarmigan survey data were available for the years from 2009 through 2023. In Grong municipality, ptarmigan line transect surveys were not conducted annually. Lierne is a neighbouring municipality to Grong and since ptarmigan densities tend to be spatially autocorrelated, we used ptarmigan densities from Lierne to extrapolate for area B (Kvasnes et al. 2014). In Meråker the line transect survey data were available from 2013 onwards, and for Røyrvik from 2018 onwards.

#### 2.2.4 Weather Data

We obtained data on weather variables via the Norwegian Meteorological Institute, using the SeNorge model with the GridTimeSeries API request (Lussana et al. 2018), which provides gridded weather data with a resolution of 1 × 1 km. For each of the nest coordinates in our dataset we extracted daily mean values for temperature (°C), snow depth (cm) and precipitation (mm). In this dataset, precipitation can fall either as snow or rain depending on the air temperature, which means that snowfall is also included in precipitation. For the productivity analysis, we calculated annual means for each of these variables over the period between 15 May and 1 July, which approximately represents the nestling period. For the occupancy analysis, we calculated the annual average values for February, because during this month we are certain that gyrfalcons are present in their territories, and we expect their territorial behaviour to be at its peak (B.C. Moen, pers. comm.). As a proxy for the timing of spring, we used the snow depth on 20 May (SD20) each year for each of the nest locations, i.e., day 140 in Figure 1e–g. Based on this, and observations from our study area, we assume that the amount of snow on 20 May reflects well how long it will take for the snow to disappear from the ground. We expect this snow level to be highly correlated to the snow level several days before and after 20 May. The presence of snow is important for the onset of activity of the flora and fauna in spring (Cooper 2014), so we assume that more snow on 20 May implies a late onset of spring, and vice versa.

## 3 Statistical Analyses

All statistical analyses were done using R version 4.4.0 in R Studio (R Core Team 2024). An overview of all the models and their structure is shown in Table 1. Rather than pursuing the minimal adequate model, all predictor variables we considered relevant were included in the models following Mundry and Nunn (2009). Interpretation and reporting of the model results were done following Muff et al. (2022), using a combination of p‐values and ‘language of evidence’. Model assumptions were evaluated through visual inspection of plots from the *DHARMa* package (Hartig 2022), to check over‐ or under dispersion, uniform distribution of the residuals, limit heteroscedasticity and outliers in the model simulations for the linear mixed models, and normally distributed random effects for the generalised linear mixed models. We did not detect multicollinearity or problematic pairwise correlations in any of the models. Multicollinearity was checked by calculating variance inflation factors (VIF) using the *car* package (Fox and Weissberg 2019) in R, with a threshold value of 5. Pairwise correlations among covariates were visualised in a correlation matrix using the *corrplot* package (Wei and Simko 2021), applying a maximum correlation coefficient threshold of 0.7.

**TABLE 1:** Overview of all the models used throughout the analyses.

Model number	Response variable	Transformed	Distribution	Description	Predictor variable(s)	Sample size	Random effect
**Diet & Feeding behaviour**							
1	Proportion ptarmigan in the diet	No	Binomial	Indicates whether prey brought into the nest is ptarmigan or alternative prey	Julian day, sex, snow depth, temperature, precipitation, SD20 *	1062	Nest‐year
2	Total time spent feeding	Square root	Gaussian	Sum of the length of all feeding events per day, per nest, per parent	Nestling age, sex, temperature, precipitation, brood size	321	Nest‐year
3	Mean length of feeding	Square root	Gaussian	Mean length of a single feeding event per day, per nest, per parent	Nestling age, sex, temperature, precipitation, brood size	321	Nest‐year
4	Prey delivery rate	No	Poisson	Sum of prey delivery events per day, per nest, per parent	Nestling age, sex, snow depth, temperature, precipitation, brood size	470	Nest‐year
**Functional response**							
5	Weekly kill rate	No	Gaussian	The mean number of ptarmigan killed per week, per nest	Ptarmigan density	42	Year
**Numerical demographic response**							
6	Combined annual productivity	No	Poisson	The number of nestlings produced per area, per year	Ptarmigan density, snow depth, temperature, precipitation, SD20	44	Area
7	Individual reproductive success	No	Poisson	The number of nestlings produced per nest, per year	Ptarmigan density, snow depth, temperature, precipitation, SD20	221	Area
**Numerical aggregative response**							
8	Territory occupancy	No	Binomial	Binary variable indicating whether a nest site is occupied or not	Ptarmigan density autumn, snow depth, temperature, precipitation, SD20	312	Area
9	Territory occupancy	No	Binomial	Binary variable indicating whether a nest site is occupied or not	Ptarmigan density preceding autumn, snow depth, temperature, precipitation, SD20	305	Area
10	Territory occupancy	No	Binomial	Binary variable indicating whether a nest site is occupied or not	Ptarmigan density autumn 2 years ago, snow depth, temperature, precipitation, SD20	300	Area
11	Territory occupancy	No	Binomial	Binary variable indicating whether a nest site is occupied or not	Ptarmigan density autumn 3 years ago, snow depth, temperature, precipitation, SD20	297	Area

*Note:* Models are divided into the different components of the statistical analysis. In two cases the response variable was transformed to adhere to the modelling assumptions. Model numbers are used for reference in the main text. Random factor nest‐year is a combination of the nest location and the year of the breeding attempt.

* SD20 = Snow depth on 20 May.

### 3.1 Diet and Feeding Behaviour

We analysed the diet and feeding behaviour of gyrfalcons based on the nest picture data using linear mixed models and generalised linear mixed models, using the *lme4* package in R (Bates et al. 2015). We set up five models with different response variables that each included similar sets of covariates. Julian day and nestling age showed a correlation coefficient higher than 0.7, so we did not include them together in the same model. Instead, we used AIC selection to determine which of these measures for time gave a better model fit. An overview of the models and descriptions of each of the response variables used for this part of the analysis are shown as models 1–4 in Table 1. With model 1, we describe the species of prey that are brought into the nest. Because we consider only freshly delivered prey here, deliveries of cached prey are excluded from these models. We expect that phenology can play a role in prey selection, as prey availability changes throughout the breeding period. In our models, phenology is reflected by Julian day or nestling age and SD20. We also expect weather, i.e., temperature, precipitation, and snow depth, to play a role in the ability of the gyrfalcons to capture certain prey types, because the visibility of prey may change in varying weather conditions. We expect that females are able to capture larger or heavier prey species because of their larger size. Models 2, 3, and 4 are related to feeding behaviour. We expect this to change as the nestlings get older, since their behaviour and energetic requirements change with their development. As documented before in gyrfalcons and other raptors, we predict that the female invests more in feeding the nestlings compared to the male. We predict that weather can play a role in the frequency of prey deliveries and may cause a changing balance between spending time feeding nestlings and hunting for prey away from the nest. We included snow depth in model 4 because the amount of snow may affect the visibility of certain prey types. In each of the models, nest‐year is included as a random effect. Our data did not allow us to include two random effects for ‘year’ and ‘nest’ simultaneously, because this led to model singularity. Nest‐year indicates the nest location and the year of the breeding attempt, for example, ‘A_23’ for nest location A in 2023, so including this as a random effect ensures we consider the variation between individual breeding attempts. We tried to incorporate a model for prey biomass, but the modelling assumptions were not met, and adjusting the model structure did not resolve these issues. Details about methods and interpretation of this model are not discussed here but can instead be found in Supporting Information S1.

### 3.2 Functional Response

The functional response was analysed by fitting the data to a type II curve, a type I curve, and an intercept‐only model. The model for the type II functional response curve was based on Hollings disc equation (Holling 1959), but the interpretation of the parameters followed the methods described by Nilsen et al. (2009). The formula is as follows:

$$y=\frac{\mathrm{ax}}{h+x}$$

Here, *y* is the kill rate as a function of prey density *x*, *a* is the asymptotic kill rate (i.e., the maximum kill rate) and *h* is the half saturation density (i.e., the ptarmigan density where 0.5*a* is reached). We calculated the kill rate as the number of ptarmigan brought into each individual nest, sensu Nyström et al. (2005), for each week in the dataset (*n* = 55). Deliveries of prey remains that were cached after the initial prey delivery were excluded in this part of the analysis. For incomplete weeks that only consisted of one to six days, we removed those that spanned fewer than five days (*n* = 14), and weeks with at least five days were adjusted by extrapolating the missing days to obtain a weekly kill rate (*n* = 12). We assigned the corresponding yearly ptarmigan density in Lierne to each of the weeks in our dataset and estimated the nonlinear least square estimates for a and h, using the nls function from the R *stats* package. We used ptarmigan densities from the preceding autumn because these estimates are more representative of what is available to gyrfalcons during the breeding period. To test for a type I functional response, i.e., a linear relationship in the data, we used the *lme4* package in R (Bates et al. 2015) to fit a linear mixed model with weekly kill rate as the response variable, ptarmigan density as the predictor variable, and year as a random variable (model 5, Table 1). To assess their fit to our data, we calculated AIC and R^2^ for both models and an intercept‐only model. We calculated R^2^ by using ordinary least squares (OLS) regression, comparing the variance explained by the model to the total variance in the data. Due to the relatively low sample size and, therefore, lack of variation in the data, we did not have sufficient statistical power to estimate the effects of weather on the functional response or kill rate.

### 3.3 Numerical Response

We divided the numerical response into two distinct components: the numerical demographic response relating to productivity, and the numerical aggregative response relating to territory occupancy, both assessed for the years 2012 through 2023. Since we do not know exactly which nests belong to which territory in our study area, we will discuss nest occupancy rather than territory occupancy hereafter. For the productivity analysis, we considered two generalised linear mixed models with the same structure but a different response variable, using the *lme4* package in R. The first is termed combined productivity, which is the sum of nestlings produced in each area (see Figure 1) per year, i.e., the combined effect of nest occupancy and reproductive success for each area. When combined productivity is higher, this means the total number of nestlings is higher, which is related to more occupied nests in this area. The second is individual reproductive success, as the number of nestlings produced per nest. We related these two response variables to ptarmigan density from the preceding autumn and weather, as shown in models 6 and 7 (Table 1). Observations were only included when we were confident about the number of nestlings that were produced at a nest (*n* = 221). Instances of lower confidence include an unreliable estimate of brood size due to, for example, bad weather conditions or a restricted view due to vegetation. For the occupancy analysis, we included all observations where nest occupancy was assessed (*n* = 319). To account for a potential time lag in the response of the gyrfalcons in territory occupancy, we calculated a ‘time‐lagged’ ptarmigan density for each observation for the same location the year before (t‐1), two years before (t‐2) and three years before (t‐3). Since ptarmigan transect data was not available before 2013 for Meråker, and before 2018 for Røyrvik, a time lag could not be considered for these years and locations. We set up four generalised linear mixed models where territory occupancy is always the response variable, and ptarmigan density with 1–3 year time lags are response variables, together with weather variables for the month of February, see models 8 to 11 (Table 1). To correct for temporal autocorrelation in territory occupancy, we incorporated an autoregressive correlation structure into these models using the ar1 function from the *glmmTMB* package (Brooks et al. 2017).

## 4 Results

### 4.1 Diet

**FIGURE 2:**
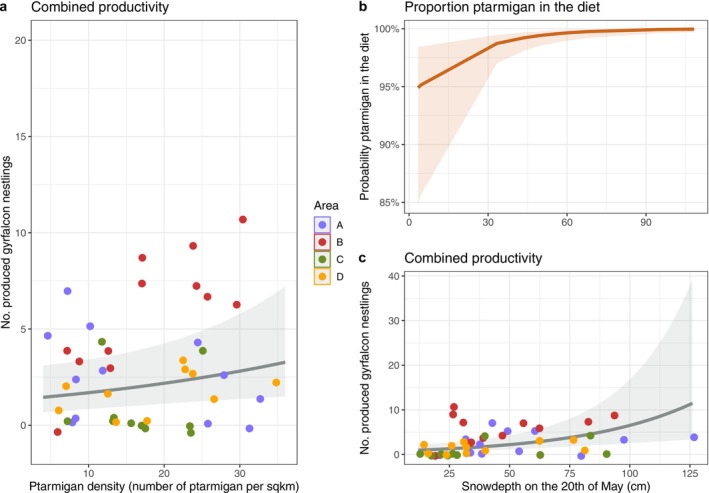
Model predictions (solid line) and their 95% confidence intervals (shaded ribbons). Left: (a) The relationship between ptarmigan density and gyrfalcon reproductive success measured as number of nestlings produced per area, i.e., combined productivity. Right: (b) The relationship between spring timing, measured as snow depth on 20 May, and the proportion ptarmigan in the diet (c) The relationship between spring timing and combined productivity. For combined productivity, raw data points are shown in different colours for the areas A–D, which is the random factor in these models.

By monitoring 12 gyrfalcon nests during the nestling periods of 2018 through 2023, we documented 1062 prey deliveries (when excluding 51 cached deliveries, Table S1). Before imputation, 863 prey deliveries (81.4%) were identified as ptarmigan, which increased to 1038 (97.7%) after imputation of unknown prey items (*n* = 175). The logistic regression model used for imputation showed good discriminatory power, with an AUC of 0.81. A complete overview of the model results related to diet and feeding behaviour can be found in Table S2. The proportion of ptarmigan in the diet decreased later in the season (odds ratio (OR) = 0.92, CI: 0.87–0.99, *p* = 0.017). When spring was later, indexed by SD20, the proportion of ptarmigan in the diet was greater (OR = 1.05, CI: 1.01–1.09, *p* = 0.010, Figure 2b). We found no evidence for a relationship between the proportion of ptarmigan in the diet and mean daily snow depth (*p* = 0.196), temperature (*p* = 0.334) or precipitation (*p* = 0.212). We found weak evidence that males brought in a lower proportion of ptarmigan than females (OR = 0.33, CI: 0.01–1.12, *p* = 0.074).

### 4.2 Feeding Behaviour

We recorded a total of 318 feeding events, with a mean feeding time of 40.6 (SE: ± 1.84) minutes per nest per day. On average, we recorded 78.5 prey deliveries per nest in 2018, 116.5 prey deliveries in 2019, 80.5 in 2020, 86.3 in 2021, 66 in 2022, and 92.5 in 2023. When the nestlings became older, the parents spent less time feeding them per day (β = −0.21, CI: −0.24 to −0.18, *p* < 0.001), the average length of a feeding event decreased (β = −0.07, CI: −0.09 to −0.06, *p* < 0.001), and the number of prey deliveries decreased (IRR = 0.98, CI: 0.97–0.99, *p* < 0.001). Males spent less time feeding nestlings per day than females (β = −2.61, CI: −3.10 to −2.12, *p* < 0.001), showed shorter feeding events (β = −0.29, CI: −0.55 to −0.04, *p* = 0.025) and brought prey to the nest less frequently (IRR = 0.51, CI: 0.42–0.61, *p* < 0.001). The rate of prey deliveries increased when brood size was larger (IRR = 1.15, CI: 1.03–1.28, *p* = 0.015), and the time spent feeding per day increased with brood size (β = 0.94, CI: 0.48–1.41, *p* < 0.001). Brood size was not related to the mean length of a feeding event (*p* = 0.144). The mean length of feeding slightly increased with temperature (β = 0.03, CI: 0.002–0.05, *p* = 0.036), but we did not find evidence for a relationship between weather variables and time spent feeding per day (temperature: *p* = 0.557, precipitation: *p* = 0.906), mean feeding time and precipitation (*p* = 0.810) or the prey delivery rate and weather variables (snow depth: *p* = 0.199, temperature: *p* = 0.154, precipitation: *p* = 0.298).

### 4.3 Functional Response

Mean weekly kill rate of ptarmigan fluctuated between 11.0 and 37.0 individuals per nest, with an overall mean of 22.53 (SE: ± 0.95). However, across the range of ptarmigan densities observed in our study [range: 17.1 to 30.4 ptarmigan per km^2^], we did not find any clear evidence that the kill rates increased with increasing ptarmigan density, as the intercept‐only model (AIC: 274.50) was equally supported by the data as both a type I (ΔAIC: 0.26) and type II functional response (ΔAIC: 0.14). The estimated parameters for the type II functional response were 31.31 (SE: ± 9.31) and 9.47 (SE: ± 10.12) for a and h, respectively. For the type I functional response, the slope was estimated at β = 0.30 (CI: −0.26‐0.86, *p* = 0.38).

### 4.4 Numerical Response

From spring 2012 through summer 2023, we recorded 55 gyrfalcon nesting attempts that produced at least one nestling. We found moderate evidence that the combined productivity, i.e., total reproductive success per area, was positively related to ptarmigan density (IRR = 1.03, CI: 1.00–1.05, *p* = 0.043, Figure 2a) and strong evidence that combined productivity increased when spring arrived later (IRR = 1.02, CI: 1.00–1.04, *p* = 0.002, Figure 2c). We found moderate evidence that combined productivity was negatively related to average snow depth during the breeding period (IRR = 0.99, CI: 0.98–1.00, *p* = 0.015). Annual reproductive success per nest increased when spring arrived later (IRR = 1.02, CI: 1.01–1.03, *p* = 0.001), but showed a negative relationship with average snow depth during the breeding period (IRR = 0.99, CI: 0.98–1.00, *p* = 0.003). We did not find clear evidence for a relationship between ptarmigan density and reproductive success per nest per year (IRR = 1.02, CI: 0.99–1.04, *p* = 0.156).

Of the 319 nest observations between 2012 and 2023, nest sites were occupied 84 times. Our findings did not reveal a correlation between the occupancy of nests and ptarmigan density, regardless of whether we considered a time lag of one, two, or three years (same year: *p* = 0.904, t‐1: *p* = 0.722, t‐2: *p* = 0.748, t‐3: *p* = 0.953, Table S3). Nest occupancy was not influenced by mean temperature and precipitation in February (*p* > 0.1, Table S3), but for each considered time lag, there was weak evidence for a positive relationship between nest occupancy and mean snow depth in February (all: β = 0.004 same year: *p* = 0.073, t‐1: *p* = 0.087, t‐2: *p* = 0.091, t‐3: *p* = 0.090).

## 5 Discussion

### 5.1 Gyrfalcons as Ptarmigan Specialists

In accordance with previous studies on gyrfalcon diet in central (Hagen 1952; Langvatn and Moksnes 1979) and northern Scandinavia (Dementiev and Gortchakovskaya 1945; Huhtala et al. 1996; Koskimies and Sulkava 2011; Lindberg 1984; Nyström et al. 2006), we found that ptarmigan is the main prey species of gyrfalcons in central Norway in the breeding season. During the breeding period, migratory bird species increase in abundance in gyrfalcon territories, and rodents become more readily available. Accordingly, some studies have shown an increase in alternative prey in the diet later in the breeding season (Potapov 2011; Robinson et al. 2019). Despite a substantial increase in prey diversity (Mellard et al. 2019), we only observed a minor increase of alternative prey in the diet as the breeding season progressed, indicating that adult gyrfalcons in central Norway specialise in ptarmigan throughout the breeding season. It is likely that ptarmigan remains the dominant prey in the gyrfalcon diet for the rest of the year, as it is the only prey species in its weight class that is present throughout the winter. It would be valuable to verify this, but our cameras only provided diet and feeding behaviour data from the breeding period.

Sometimes it was challenging to confidently identify the species of a prey based on the camera data, which resulted in ~16% unknown prey. Lack of background knowledge on the current gyrfalcon diet in our study area made it challenging to estimate the real underlying proportions. When the abundance of alternative prey in the area increases during the monitoring period, missing prey items are more likely to be alternative prey rather than ptarmigan. Alternative prey is often smaller, making them harder to identify. However, ptarmigan are generally more heavily processed before reaching the nest, e.g., plucked, decapitated, or split in parts, which would make alternative prey easier to identify in such cases. We have addressed this by taking the increasing availability of alternative prey into account by including nestling age and snow depth as covariates for phenology in the imputation model and potential weather effects on hunting success by including temperature and precipitation.

We also investigated gyrfalcon feeding behaviour, and our findings align well with results from previous studies. Female birds invest more time in feeding their nestlings and delivery and feeding rates increase with increasing brood size (Booms and Fuller 2003b; Sonerud et al. 2014). A decline in feeding rates as nestlings get older may be explained by a reduced energetic need. Models on avian development show that gyrfalcon nestlings are at the peak of their energetic consumption when they are 15–20 days old (Konarzewski et al. 1998; Weathers 1992). This coincides with installation of the nest cameras when nestlings are 10–20 days old and may explain the reduced feeding rates. However, we cannot verify this using our data because we lack data from the early nestling phase. Besides, close to fledging, nestlings sometimes move away from the nest where they may still be fed by their parents. It is possible that we have missed such feeding events and prey deliveries outside of camera view at the end of the monitoring period, which may lead to the observed decline in feeding rates. Another limitation of the camera monitoring is that we do not have consistent data on behaviour of the parents away from the nest. It is clear that females spend more time feeding nestlings compared to males, but males may be responsible for the capturing of the prey. Gyrfalcons are known to have ‘plucking spots’ where prey is processed before it is delivered to the nest (Booms et al. 2020). Exchange of prey between male and female might take place here, so we cannot conclude from our results that females invest more in parental care. We observed no impact of weather variables on feeding behaviour, which suggests that reproductive success is primarily influenced through prey availability or hunting success, rather than by changing feeding behaviour.

For specialist predators like the gyrfalcon, a type II functional response is expected (Holling 1966), though in our study we found no clear evidence that our data fit a type I or type II functional response. Nyström et al. (2005) revealed a type II functional response of gyrfalcons to ptarmigan density in Sweden, and Nielsen (1999) found evidence for both a type I and type II functional response in Iceland. Since kill rates are challenging to calculate for wild populations, sample sizes are often low for such studies (Dale et al. 1994; Nilsen et al. 2009). It is likely that ptarmigan densities differ slightly between gyrfalcon territories, depending on the landscape and vegetation. In our analysis, we calculated the same yearly ptarmigan densities for all nests within an area. More specific density estimates would require more specific knowledge of the territory characteristics and different counting and estimation methods. Besides, the fluctuations in ptarmigan density in our dataset were relatively moderate. A lack of variation in ptarmigan densities may explain why we did not observe a functional response. It should also be noted that we used ptarmigan density estimates from the preceding autumn because counts from the breeding period itself were not available. The studies that did find a functional response either estimated ptarmigan densities in the spring (Nielsen 1999) or summer (Nyström et al. 2005), which may be more accurate than our counts from the preceding autumn. There is a peak of mortality in the autumn for ptarmigan in Norway due to a combination of harvest and natural mortality, but a natural mortality peak also occurs in spring (Eriksen et al. 2025). If these patterns are not consistent across years, it may reduce the accuracy of our estimates and, consequently, our ability to predict the effect of ptarmigan density on gyrfalcon feeding rates.

Corresponding to our expectations and previous studies (Barichello and Mossop 2011; Falkdalen et al. 2011; Hörnell‐Willebrand 2008; Nyström et al. 2005), we found a positive numerical demographic response. This relationship is most pronounced when examining combined productivity per area but becomes much weaker, to non‐existent, when considering individual nesting success. The numerical aggregative response has not reached consensus in prior studies (Hörnell‐Willebrand 2008; Nielsen 1999; Nyström et al. 2005; Selås and Kålås 2007). ‘Combined productivity’ is a measure that combines territory occupancy and individual reproductive success. In our study with our sample size, it appears that territory occupancy and reproductive success individually do not show sufficient variation to respond to fluctuating ptarmigan density. However, when combined they do show a correlation with ptarmigan density (Figure 2a), suggesting that bottom‐up control can be an important factor in gyrfalcon population dynamics. Similarly, using ptarmigan density estimates from the preceding autumn rather than from the breeding period itself may lead to an underestimation of the strength of the relationship between ptarmigan density and gyrfalcon productivity and nest occupancy.

Despite our thorough monitoring efforts of gyrfalcon nesting locations, there may be some limitations to the data collection methods used during the surveys. Monitoring is generally limited to known nest locations, so we may underestimate gyrfalcon nest occupancy if they occupied unknown nest sites. We believe this to be a minor issue, because there are few suitable cliffs for nesting gyrfalcons in our study area, and any surrounding cliffs are also surveyed for potential new nest locations. We could have missed occupancy of nests that failed before the occupancy surveys and showed no obvious signs of previous gyrfalcon occupation. This could result in an underestimation of nest occupancy and provides an alternative explanation for the lack of a numerical aggregative response. Underestimating gyrfalcon nest occupancy may obscure the response to fluctuating ptarmigan densities. If such nests failed before our surveys due to low ptarmigan densities, this could have also enhanced the numerical demographic response that we observed, because late surveys likely lead to an overestimation of successful breeding attempts. Additionally, we may miss nestlings that have already fledged during the counts, and nestlings we assume will fledge may still perish after our surveys. The former should only be an issue if there is significant asynchrony in the timing of nests, since surveys are generally conducted when nestlings still have down on their bodies and are not yet ready to fully fledge. It is also uncommon for nestlings to die during this stage of their development. Finally, our data was too limited to account for observation bias, preventing us from evaluating whether factors like time of day or specific weather conditions influenced the detection of occupancy and the counting of nestlings. This means that, for example, the effect of weather on gyrfalcon reproduction and nest occupancy could be underestimated in our study.

### 5.2 Late Springs Benefit Gyrfalcon Reproductive Success

Our study shows contrasting effects of snow depth on gyrfalcon productivity between different phases of the breeding period. In accordance with a study from Iceland (Nielsen 2011), we found evidence that average snow depth during the nestling period is negatively correlated to both combined productivity and individual reproductive success. In addition to these findings, our study revealed that gyrfalcon breeding is more successful when spring is late (i.e., more snow around hatching of the nestlings), that the proportion of ptarmigan in their diet is higher in later springs, and that territory occupancy seems positively related to snow depth during territory establishment in February. In other words, more snow is generally beneficial during the early parts of the breeding season, but has a negative effect when nestlings get older. This may not be surprising and suggests that gyrfalcons are well adapted to traditional winter conditions in the mountains, where snow is naturally abundant in winter and spring, but melts throughout the development of the nestlings. Our findings can be complemented by another study from our study area that revealed that ptarmigan mortality is higher in springs with more snow (Eriksen et al. 2025). We propose a few potential interpretations for these findings. Firstly, we speculate that the camouflage mismatch theory could play a role when the transition from white to brown plumage of ptarmigan does not align with the timing of snow melt (Imperio et al. 2013; Otte et al. 2024; Zimova et al. 2020). This makes ptarmigan more conspicuous and an easy target for predators such as the gyrfalcon, but the same could also happen in very early springs (Melin et al. 2020). The period of snow melt generally coincides with the ptarmigan courtship period, when males perform vocal and visual displays to attract females. This seasonal behaviour makes them more visible during this period compared to the summer, enhancing a potential camouflage mismatch (Nielsen and Cade 2017). Secondly, in late springs ptarmigan may be forced to feed on the few snow‐free patches where the vegetation is accessible, but they are more exposed to predation (Steen et al. 1992). Additionally, due to higher energetic requirements during nesting but lower diet quality with much snow cover (García‐González et al. 2016; Rixen et al. 2022), ptarmigan may also need to spend more time feeding on these few patches when further snow melt is delayed. Finally, in years with late springs, alternative prey species such as migratory birds and rodents are expected to become available later in gyrfalcon territories. With fewer alternative prey available, gyrfalcons are forced to rely even more heavily on ptarmigan, resulting in higher ptarmigan proportions in the gyrfalcon diet and higher mortality rates for ptarmigan. This is likely related to increased gyrfalcon breeding success and territory occupancy, since ptarmigan seem to be their favoured and optimal prey species (Nielsen and Cade 1990).

### 5.3 Predators in a Changing World

In many areas, the onset of spring is advancing due to the warming climate (Inouye 2022; Parmesan and Yohe 2003), which may lead to ecological mismatches. This can have significant consequences across natural ecosystems, especially at high latitudes. Migratory raptors are forced to advance their arrival to their breeding grounds to synchronise reproduction with the peak of food availability. Successful adaptation has been associated with higher breeding success, while an unsuccessful advancement may result in a phenological mismatch, reducing breeding success (Martinez‐Ruiz et al. 2023). The same applies to range shifts in mammalian predators such as the lynx (van Hassel and Bovenkerk 2023) and wolverine (McKelvey et al. 2011) that need to adapt to maximise the potential for successfully hunting prey. Our study demonstrates that late springs are associated with increased gyrfalcon breeding performance, likely due to the changing availability of ptarmigan under varying snow conditions. In this context,' availability' encompasses not only abundance but also visibility and other factors influencing the gyrfalcon's hunting success, which also vary with environmental conditions. Given that these changes in prey availability due to shifting phenology may lead to decreased breeding success under the current climate predictions, gyrfalcons may need to adapt in the future.

Climate warming can affect small herbivores such as ptarmigan both positively and negatively. Positive effects of earlier springs include increasing food and nest availability, more shelter from vegetation cover, and higher offspring survival in milder temperatures (Eriksen et al. 2023; Findlay‐Robinson et al. 2023; Ingvaldsen et al. 2024; Layton‐Matthews et al. 2020; Morrissette et al. 2010). Processes that can impact small herbivores negatively are often observed in the long term (Fuglei et al. 2020; Ims and Fuglei 2005). An example is the northward or upslope shift of generalist predators such as the red fox ( *Vulpes vulpes* ) due to ameliorating conditions in higher altitudes and latitudes (Selås and Vik 2006). This can alter food‐web structure by increasing predation pressure on small herbivores and competition with local (specialist) predators. In addition, interconnected population cycling of mammalian populations is more prevalent in higher latitudes (Kendall et al. 2002) and has been observed for the ptarmigan and gyrfalcon populations in Iceland (Barraquand and Nielsen 2018). The food web in Scandinavia is more complex, and previous research has shown that ptarmigan population cycles are also closely linked to the rodent cycle (Hagen 1989). The alternative prey hypothesis states that in years of high rodent abundance, predation pressure is relieved for ptarmigan. It has been suggested that the attenuation of rodent cycles has led to an increase in predation pressure on ptarmigan, causing a decrease in their population size (Fuglei et al. 2020; Hjeljord and Loe 2022). This mechanism is also relevant for non‐cyclic prey, where the presence or absence of alternative prey can alleviate or enhance predation pressure for certain species, respectively (Kjellander and Nordström 2003).

The effects of climate change may differ between the two ptarmigan species in our study area. The willow ptarmigan generally occupies lower elevations with denser and taller vegetation cover (Hannon et al. 2020; Kvasnes et al. 2018), whereas the rock ptarmigan occupies higher elevations with rocky ground cover and low shrubs (Montgomerie and Holder 2020; Wilson and Martin 2008). The slightly milder habitat that willow ptarmigan inhabit is predicted to expand, potentially benefiting this species in the short term (Eriksen et al. 2023; Jackson et al. 2016). However, there are also studies that suggest that suitable habitat for both species will decline, and interspecific competition is expected to increase (Mandeville et al. 2024; Scridel et al. 2021). In Norway, the populations of willow and rock ptarmigan seem to fluctuate synchronously (Kvasnes et al. 2010). However, we are unsure about the exact relative abundance available in each gyrfalcon territory. Henderson et al. (2021) show that the habitat characteristics around gyrfalcon nests in Alaska accurately reflect the proportions of the two ptarmigan species in their diet, without showing a preference for one species. In our study we could not distinguish between the two species, so we cannot conclude whether gyrfalcons in Scandinavia are also opportunistic hunters or if they show a preference for one of the two species. Depending on this, certain gyrfalcon territories may become unsuitable for hunting in the future due to vegetation changes and corresponding changes in prey availability. To gain a deeper understanding of prey selection by gyrfalcons in Scandinavia and its implications in the context of climate change, more detailed studies on foraging behaviour and territory characteristics are needed.

The examples above illustrate that the impact of climate change is not always straightforward, and simply examining a single species is insufficient (Bowler et al. 2020; Gilman et al. 2010). Even specialist predators such as the gyrfalcon are not only directly connected to their main prey species. Indirectly, gyrfalcons are also connected to other species, because the ptarmigan is a key player in the dynamics of many other species in both lower and higher trophic levels. This study suggests that climate change will likely necessitate adaptations from gyrfalcons and other predators in alpine areas. The effects of climate change on the species predators interact with are indirect but crucial factors in assessing a predator's resilience to a changing environment. Contrasting impacts of climate change in the short‐ and long‐term on several prey species and between different habitats can further complicate these predictions. However, they demonstrate that the influence of climate change almost invariably depends on the local food‐web context.

## Author Contributions

**Annabel Josien Slettenhaar:** conceptualization (equal), formal analysis (lead), investigation (equal), methodology (lead), software (equal), validation (equal), visualization (lead), writing – original draft (lead), writing – review and editing (lead). **Jan Eivind Østnes:** conceptualization (equal), formal analysis (supporting), funding acquisition (equal), investigation (equal), project administration (equal), supervision (equal), writing – original draft (supporting), writing – review and editing (equal). **Børje Cato Moen:** data curation (equal), investigation (equal), resources (equal), writing – review and editing (equal). **Rolf Terje Kroglund:** investigation (equal), resources (equal), writing – review and editing (equal). **Torgeir Nygård:** data curation (equal), investigation (equal), resources (equal), writing – review and editing (equal). **Erlend Birkeland Nilsen:** conceptualization (equal), formal analysis (supporting), funding acquisition (equal), investigation (equal), methodology (supporting), project administration (equal), resources (equal), supervision (equal), validation (equal), visualization (supporting), writing – original draft (supporting), writing – review and editing (equal).

## Conflicts of Interest

The authors declare no conflicts of interest.

## Supporting information


Data S1.

**Table S1.** Prey items delivered to the nests in Lierne municipality between 2018 and 2023.

**Table S2.** Results from (generalised) linear (mixed) models describing gyrfalcon diet and feeding behaviour in Lierne municipality between 2018 and 2023. Depending on the type of family, odds ratio, estimates or incidence rate ratios are provided, together with corresponding confidence intervals and p‐values. P‐values in bold are below the 0.05 threshold.

**Table S3.** Results from generalised linear mixed models describing the numerical aggregative response of gyrfalcons in six municipalities in central Norway between 2012 and 2023. Odds ratios are provided with corresponding confidence intervals and p‐values.

## Data Availability

All data and R‐scripts needed to reproduce the results and figures presented in this manuscript are accessible at the Open Science Framework through the following link: https://osf.io/5zpwy/.
