# Supplementary material for: A Picky Predator and Its Prey: How Snow Conditions and Ptarmigan Abundance Impact Gyrfalcon Feeding Behaviour and Breeding Success
Source: Ecol Evol. 2025 Apr 9;15(4):e71228. doi: 10.1002/ece3.71228 (PMC11981877; doi:10.1002/ece3.71228)
Supplement: Supplementary file 1 — Data S1. [file ECE3-15-e71228-s004.pdf]

Supporting Information 1

Using the nest camera data, we wanted to investigate if and how the biomass of prey items brought into the nest was related to weather variables, if this differed between the sex of the parent, and how this changed throughout the breeding period. To this end, we assigned an average biomass to each prey species in our dataset using the AVONET dataset (Tobias et al., 2022) and panTHERIA archives (Jones et al., 2009), for avian and mammalian prey species, respectively. For prey that was classified as a group of species, we used the average biomass of several common species in that category as described in the table below. We assigned the average biomass for ptarmigan to unknown prey imputed as ptarmigan, i.e. rock ptarmigan and willow ptarmigan, and we assigned the weighted mean biomass of all alternative prey present in our dataset to unknown prey imputed as alternative prey. We used a linear mixed model with prey biomass as a response variable, and Julian day, sex of the parent delivering the prey, mean snow depth on the day of the prey delivery, mean temperature on the day of the prey delivery, and amount of precipitation on the day of the delivery as predictor variables. We included nest-year as a random effect in the model. When running the model, we detected deviations from uniformity of residuals, presence of influential outliers in model predictions and heteroscedasticity. For these reasons, we consider the results from this model unreliable, and we decided to not discuss them or include them in the main text.

Reported biomass of species used to calculate prey biomass classified as a grouped type of prey. Bird biomass is extracted from the AVONET dataset (Tobias et al., 2022) and mammal biomass from the panTHERIA archives (Jones et al., 2009).

Species	Species Latin	Reported biomass (g)
**Waders**		
Common sandpiper	*Actitis hypoleucos*	48.0
Common redshank	*Tringa totanus*	129.0
Whimbrel	*Numenius phaeopus*	364.6
Golden plover	*Pluvialis apricaria*	214.0
Wood sandpiper	*Tringa glareola*	62.1
Common greenshank	*Tringa nebularia*	187.0
**Passerines**		
Meadow pipit	*Anthus pratensis*	18.4
Willow warbler	*Phylloscopus trochilus*	8.7
Redwing	*Turdus iliacus*	61.2
Common redpoll	*Acanthis flammea*	13.0
Yellow wagtail	*Motacilla flava*	17.7
**Small rodents**		
Field vole	*Microtus agrestis*	42.5
Norwegian lemming	*Lemmus lemmus*	47.5

References

Jones, K. E., Bielby, J., Cardillo, M., Fritz, S. A., O'Dell, J., Orme, C. D. L., Safi, K., Sechrest, W., Boakes, E. H., Carbone, C., Connolly, C., Cutts, M. J., Foster, J. K., Grenyer, R., Habib, M., Plaster, C. A., Price, S. A., Rigby, E. A., Rist, J., Teacher, A., Bininda-Emonds, O. R. P., Gittleman, J. L., Mace, G. M., & Purvis, A. (2009). PanTHERIA: a species-level database of life history, ecology, and geography of extant and recently extinct mammals. *Ecology*, *90*(9), 2648-2648. https://doi.org/https://doi.org/10.1890/08-1494.1

Tobias, J. A., Sheard, C., Pigot, A. L., Devenish, A. J. M., Yang, J. Y., Sayol, F., Neate-Clegg, M. H. C., Alioravainen, N., Weeks, T. L., Barber, R. A., Walkden, P. A., MacGregor, H. E. A., Jones, S. E. I., Vincent, C., Phillips, A. G., Marples, N. M., Montaño-Centellas, F. A., Leandro-Silva, V., Claramunt, S., Darski, B., Freeman, B. G., Bregman, T. P., Cooney, C. R., Hughes, E. C., Capp, E. J. R., Varley, Z. K., Friedman, N. R., Korntheuer, H., Corrales-Vargas, A., Trisos, C. H., Weeks, B. C., Hanz, D. M., Töpfer, T., Bravo, G. A., Remes, V., Nowak, L., Carneiro, L. S., R, A. J., Matysioková, B., Baldassarre, D. T., Martínez-Salinas, A., Wolfe, J. D., Chapman, P. M., Daly, B. G., Sorensen, M. C., Neu, A., Ford, M. A., Mayhew, R. J., Silveira, L. F., Kelly, D. J., Annorbah, N. N. D., Pollock, H. S., Grabowska-Zhang, A. M., McEntee, J. P., Gonzalez, J. C. T., Meneses, C. G., Muñoz, M. C., Powell, L. L., Jamie, G. A., Matthews, T. J., Johnson, O., Brito, G. R. R., Zyskowski, K., Crates, R., Harvey, M. G., Zevallos, M. J., Hosner, P. A., Bradfer-Lawrence, T., Maley, J. M., Stiles, F. G., Lima, H. S., Provost, K. L., Chibesa, M., Mashao, M., Howard, J. T., Mlamba, E., Chua, M. A. H., Li, B. C., Gómez, M. I., García, N. C., Päckert, M., Fuchs, J. M., Ali, J. R., Derryberry, E. P., Carlson, M. L., Urriza, R. C., Brzeski, K. E., Prawiradilaga, D. M., Rayner, M. J., Miller, E. T., Bowie, R. C. K., Lafontaine, R. M., Scofield, R. P., Lou, Y. Q., Somarathna, L., Lepage, D., Illif, M., Neuschulz, E. L., Templin, M., Dehling, D. M., Cooper, J. C., Pauwels, O. S. G., Analuddin, K., Fjeldså, J., Seddon, N., Sweet, P. R., DeClerck, F. A. J., Naka, L. N., Brawn, J. D., Aleixo, A., Böhning-Gaese, K., Rahbek, C., Fritz, S. A., Thomas, G. H., & Schleuning, M. (2022). AVONET: morphological, ecological and geographical data for all birds. *Ecology Letters*, *25*(3), 581-597. https://doi.org/10.1111/ele.13898
