# Supplementary material for: A Picky Predator and Its Prey: How Snow Conditions and Ptarmigan Abundance Impact Gyrfalcon Feeding Behaviour and Breeding Success
Source: Ecol Evol. 2025 Apr 9;15(4):e71228. doi: 10.1002/ece3.71228 (PMC11981877; doi:10.1002/ece3.71228)
Supplement: Supplementary file 2 — Table S1. Prey items delivered to the nests in Lierne municipality between 2018 and 2023. [file ECE3-15-e71228-s003.pdf]

Species	Species Latin	Absolute numbers	Percentage
Ptarmigan	*Lagopus lagopus/muta*	863	81.42
Unknown		175	16.51
Whimbrel	*Numenius phaeopus*	6	0.57
Wader		4	0.38
Common gull (chick)	*Larus canus*	3	0.28
Golden plover	*Pluvialis apricaria*	2	0.19
Common scoter (chick)	*Melanitta nigra*	1	0.09
Eurasian woodcock	*Scalopax rusticola*	1	0.09
Mallard (chick)	*Anas platyrhynchos*	1	0.09
Redshank	*Tringa totanus*	1	0.09
Mountain hare (leveret)	*Lepus timidus*	1	0.09
Passerine		1	0.09
Small rodent		1	0.09
