# Supplementary material for: A Picky Predator and Its Prey: How Snow Conditions and Ptarmigan Abundance Impact Gyrfalcon Feeding Behaviour and Breeding Success
Source: Ecol Evol. 2025 Apr 9;15(4):e71228. doi: 10.1002/ece3.71228 (PMC11981877; doi:10.1002/ece3.71228)
Supplement: Supplementary file 3 — Table S2. Results from (generalised) linear (mixed) models describing gyrfalcon diet and feeding behaviour in Lierne municipality between 2018 and 2023. Depending on the type of family, odds ratio, estimates or incidence rate ratios are provided, together with corresponding confidence intervals and p‐values. P‐values in bold are below the 0.05 threshold. [file ECE3-15-e71228-s001.pdf]

Predictors	Proportion ptarmigan in diet			Time spent feeding per day (minutes)			Mean length of feeding event (minutes)			Daily prey delivery rate		
	Odds Ratios	*CI*	*p*	*Estimates*	*CI*	*p*	*Estimates*	*CI*	*p*	*Incidence Rate Ratios*	*CI*	*p*
Intercept	4157678.710	86.714 – 199348953889.494	**0.006**	9.952	8.160 – 11.745	**<0.001**	5.138	4.289 – 5.987	**<0.001**	3.675	2.249 – 6.007	**<0.001**
Julian day	0.924	0.866 – 0.986	**0.017**									
Sex, male	0.329	0.097 – 1.116	0.074	-2.610	-3.096 – -2.123	**<0.001**	-0.291	-0.545 – -0.037	**0.025**	0.508	0.423 – 0.610	**<0.001**
Sex, unknown	0.532	0.162 – 1.743	0.297	-2.075	-3.103 – -1.048	**<0.001**	-0.396	-0.933 – 0.142	0.148	0.779	0.647 – 0.937	**0.008**
Snowdepth (cm)	0.976	0.940 – 1.013	0.196							1.002	0.999 – 1.006	0.199
Temperature (°C)	1.072	0.928 – 1.237	0.344	-0.014	-0.060 – 0.033	0.557	0.026	0.002 – 0.050	**0.036**	0.989	0.975 – 1.004	0.154
Precipitation (mm)	1.163	0.917 – 1.474	0.212	-0.002	-0.033 – 0.030	0.906	0.003	-0.014 – 0.019	0.761	1.005	0.996 – 1.014	0.298
Snowdepth on 20 May (cm)	1.049	1.011 – 1.087	**0.010**									
Nestling age (days)				-0.206	-0.235 – -0.178	**<0.001**	-0.073	-0.088 – -0.058	**<0.001**	0.982	0.973 – 0.990	**<0.001**
Brood size				0.943	0.476 – 1.410	**<0.001**	0.165	-0.056 – 0.385	0.144	1.148	1.027 – 1.283	**0.015**
**Random Effects**												
σ^2	3.29			2.76			0.76			0.34		
τ_{00}	0.03_{nestID}			1.45_{nestID}			0.21_{nestID}			0.03_{nestID}		
ICC	0.01			0.34			0.22			0.08		
N	12_{nestID}			12_{nestID}			12_{nestID}			12_{nestID}		
Marginal R^2 / Conditional R^2	0.464 / 0.468			0.512 / 0.680			0.262 / 0.425			0.277 / 0.333		
