# Supplementary material for: A Picky Predator and Its Prey: How Snow Conditions and Ptarmigan Abundance Impact Gyrfalcon Feeding Behaviour and Breeding Success
Source: Ecol Evol. 2025 Apr 9;15(4):e71228. doi: 10.1002/ece3.71228 (PMC11981877; doi:10.1002/ece3.71228)
Supplement: Supplementary file 4 — Table S3. Results from generalised linear mixed models describing the numerical aggregative response of gyrfalcons in six municipalities in central Norway between 2012 and 2023. Odds ratios are provided with corresponding confidence intervals and p‐values. [file ECE3-15-e71228-s002.pdf]

	Numerical response same year			Numerical response 1-year time lag			Numerical response 2-year time lag			Numerical response 3-year time lag		
Predictors	*Odds Ratios*	*CI*	*p*	*Odds Ratios*	*CI*	*p*	*Odds Ratios*	*CI*	*p*	*Odds Ratios*	*CI*	*p*
Intercept	0.164	0.040 – 0.667	**0.012**	0.181	0.042 – 0.789	**0.023**	0.152	0.035 – 0.670	**0.013**	0.164	0.033 – 0.816	**0.027**
Ptarmigan density same year	0.997	0.954 – 1.043	0.903									
Temperature (°C)	1.012	0.875 – 1.171	0.874	1.003	0.874 – 1.150	0.969	1.009	0.874 – 1.164	0.904	1.003	0.870 – 1.156	0.968
Precipitation (mm)	0.984	0.879 – 1.102	0.777	0.991	0.881 – 1.115	0.885	0.982	0.876 – 1.100	0.750	0.983	0.878 – 1.102	0.773
Snowdepth (cm)	1.005	1.000 – 1.010	0.074	1.005	0.999 – 1.010	0.087	1.005	0.999 – 1.010	0.091	1.005	0.999 – 1.010	0.090
Ptarmigan density preceding year				0.992	0.951 – 1.035	0.722						
Ptarmigan density 2 years ago							1.007	0.967 – 1.048	0.748			
Ptarmigan density 3 years ago										0.999	0.960 – 1.040	0.953
ICC	0.00			0.00			0.00			0.00		
N	4_{area}			4_{area}			4_{area}			4_{area}		
Observations	312			305			300			297		
Marginal R^2 / Conditional R^2	0.023 / 0.023			0.021 / 0.021			0.023 / 0.023			0.021 / 0.021		
